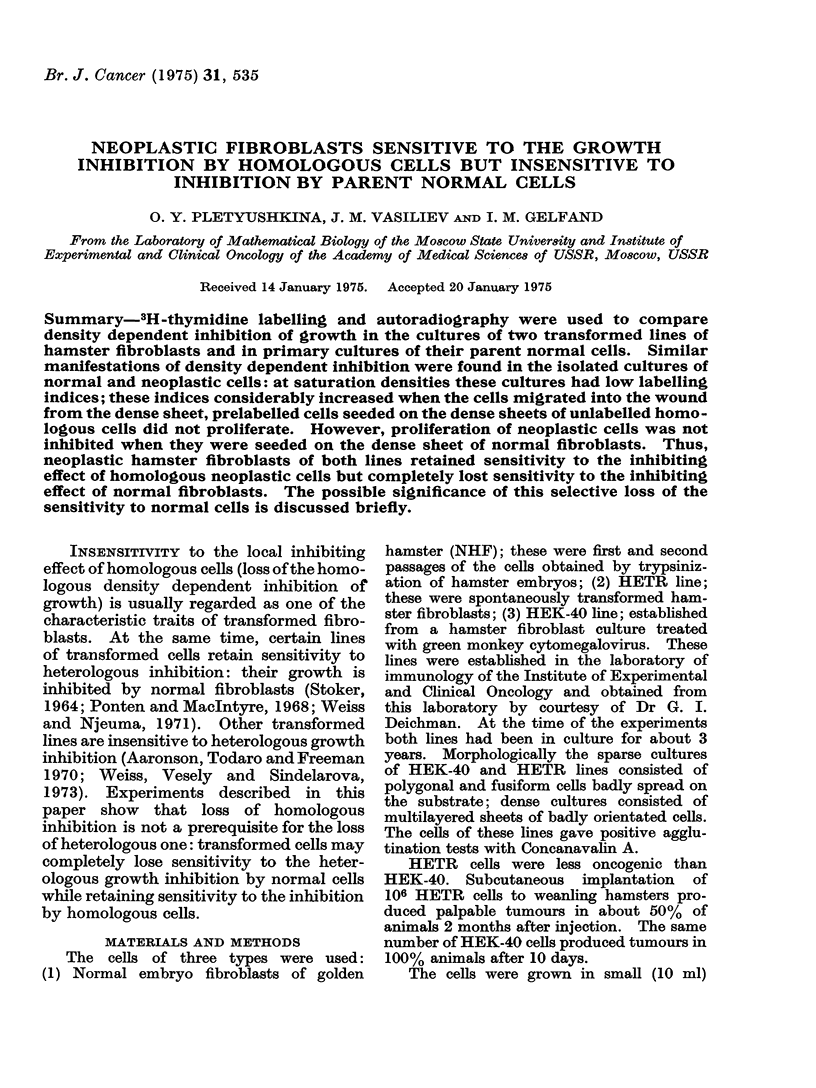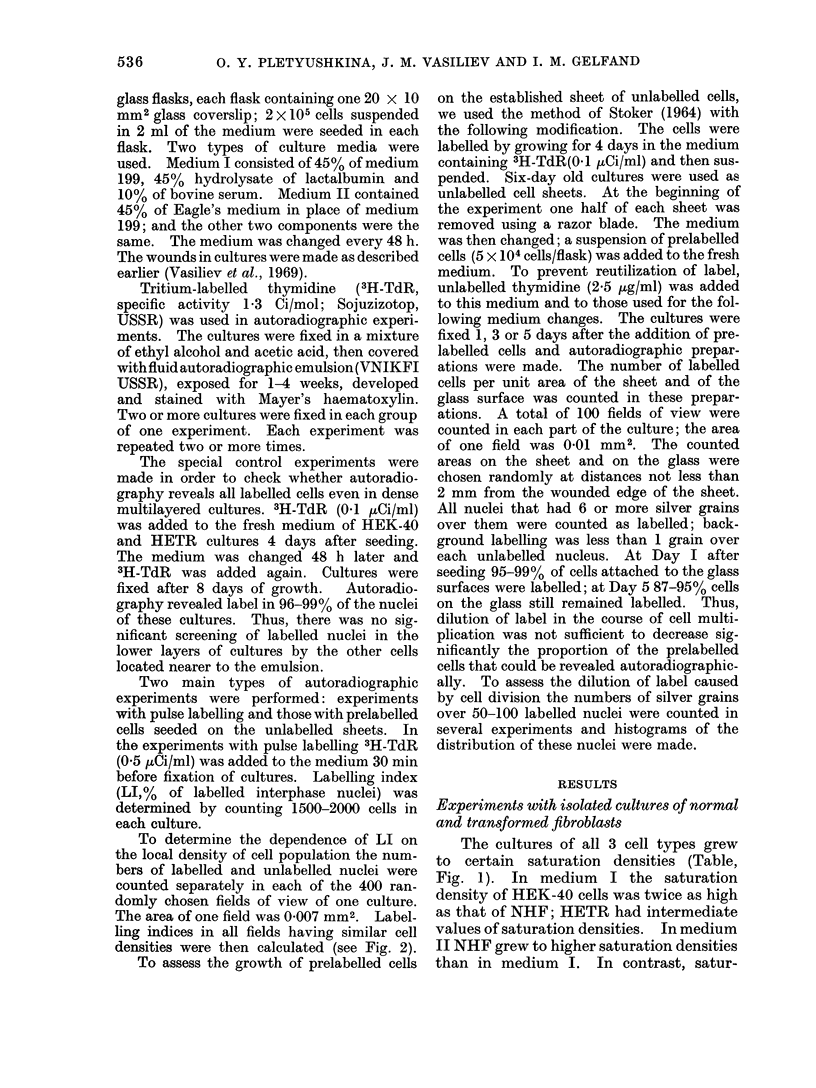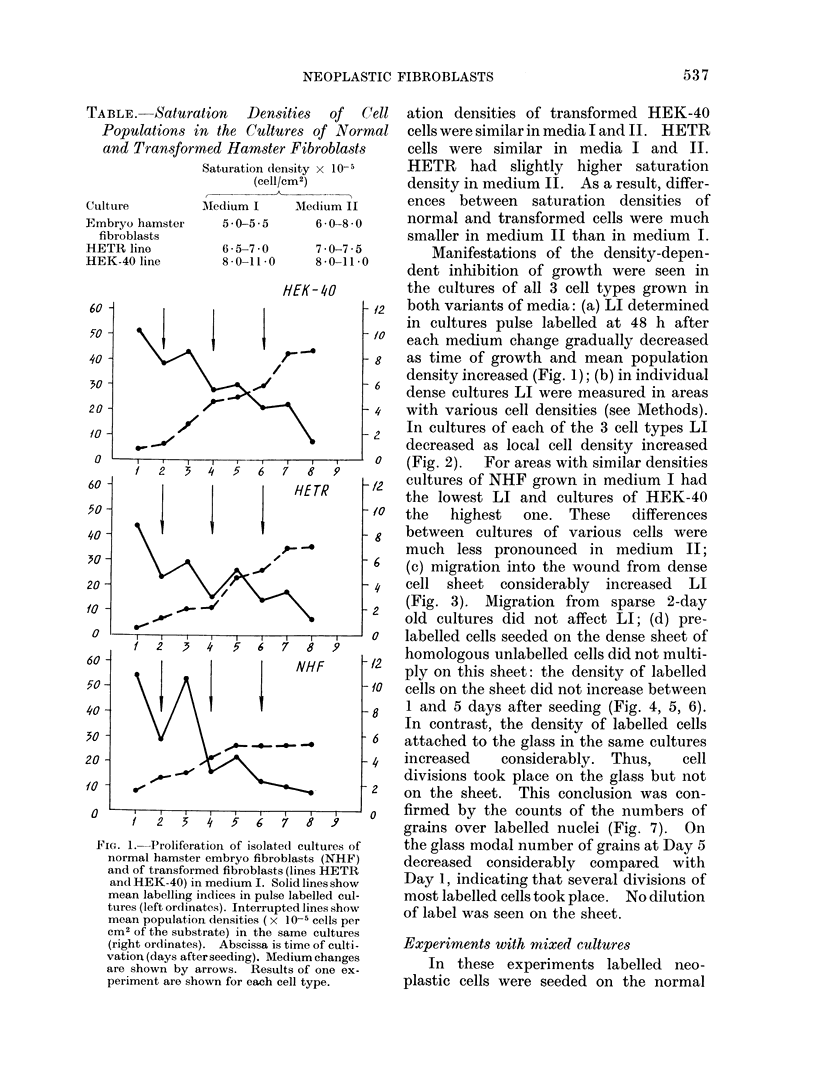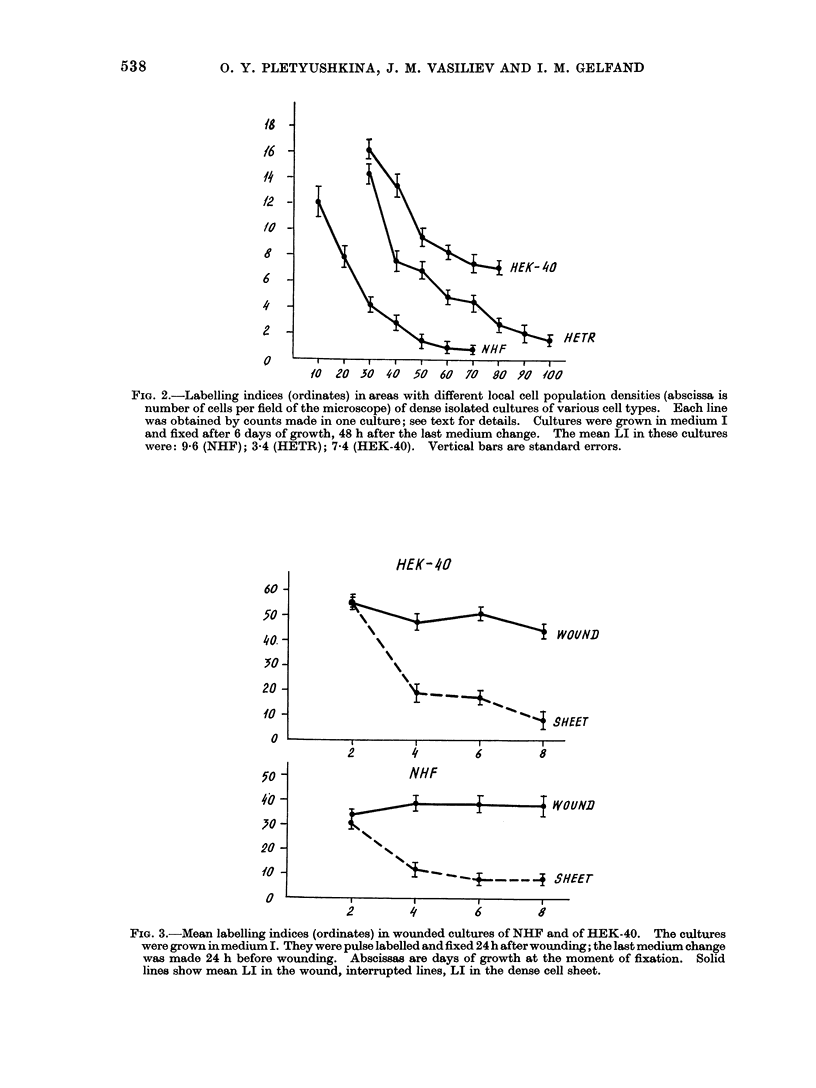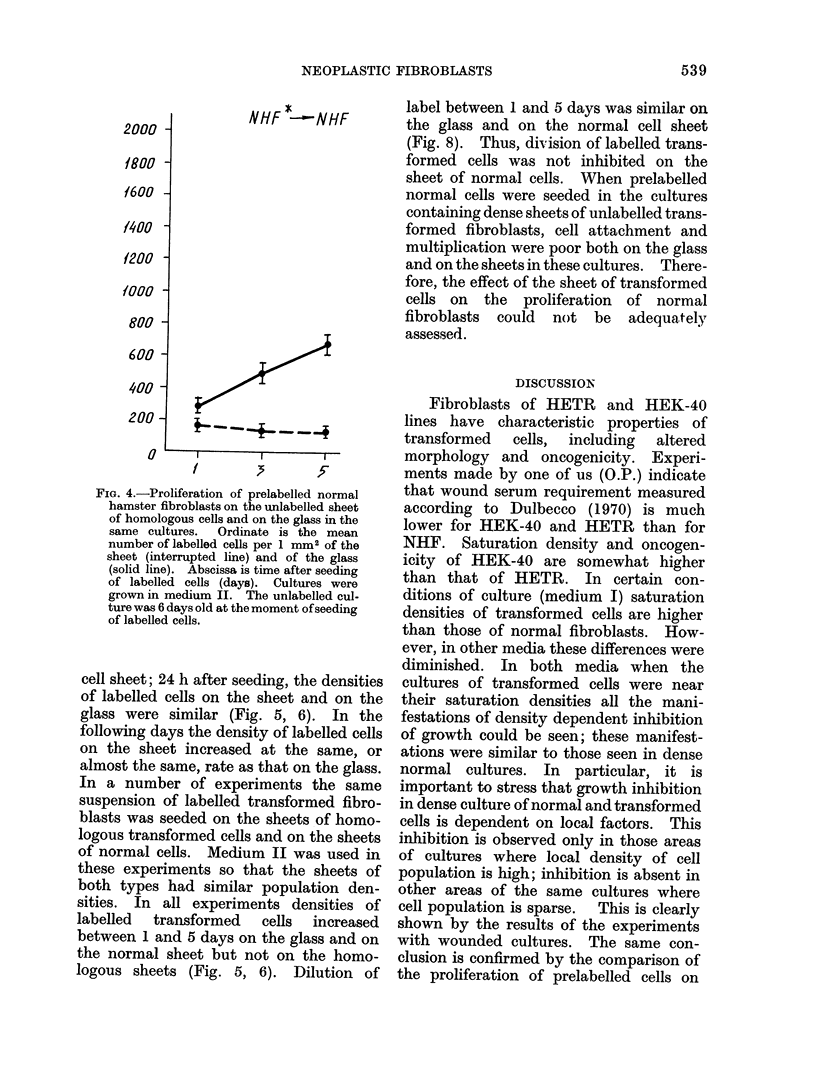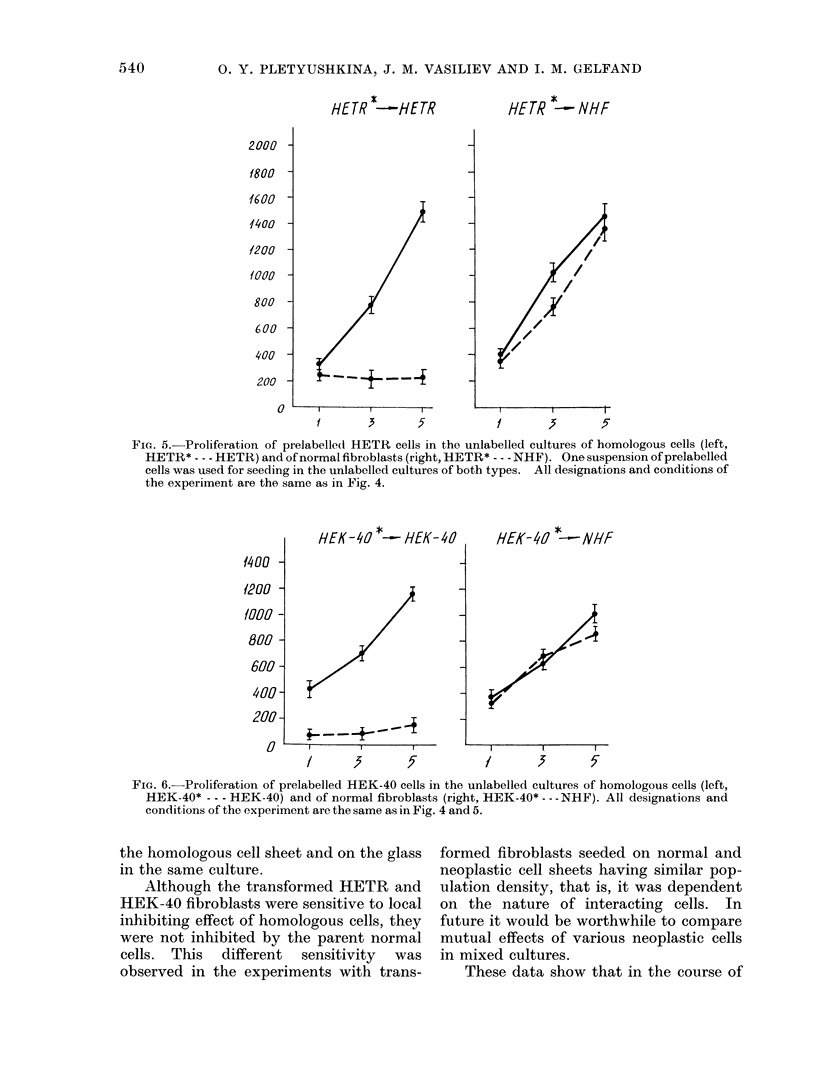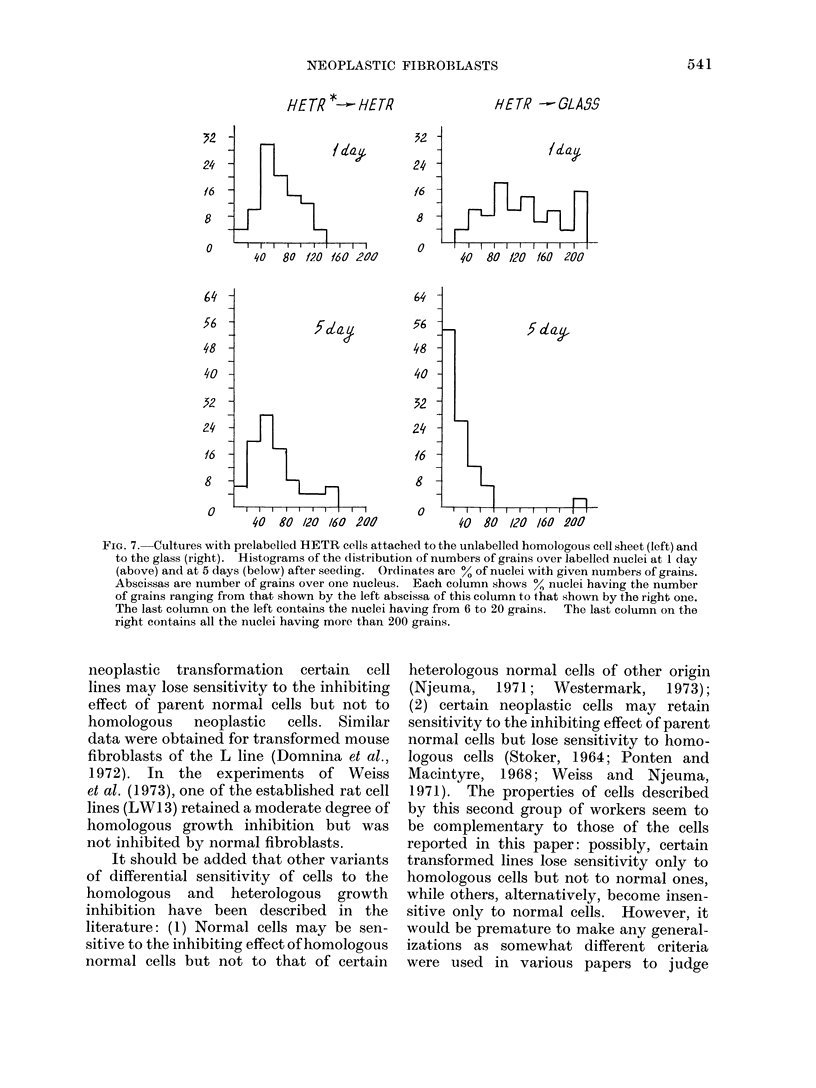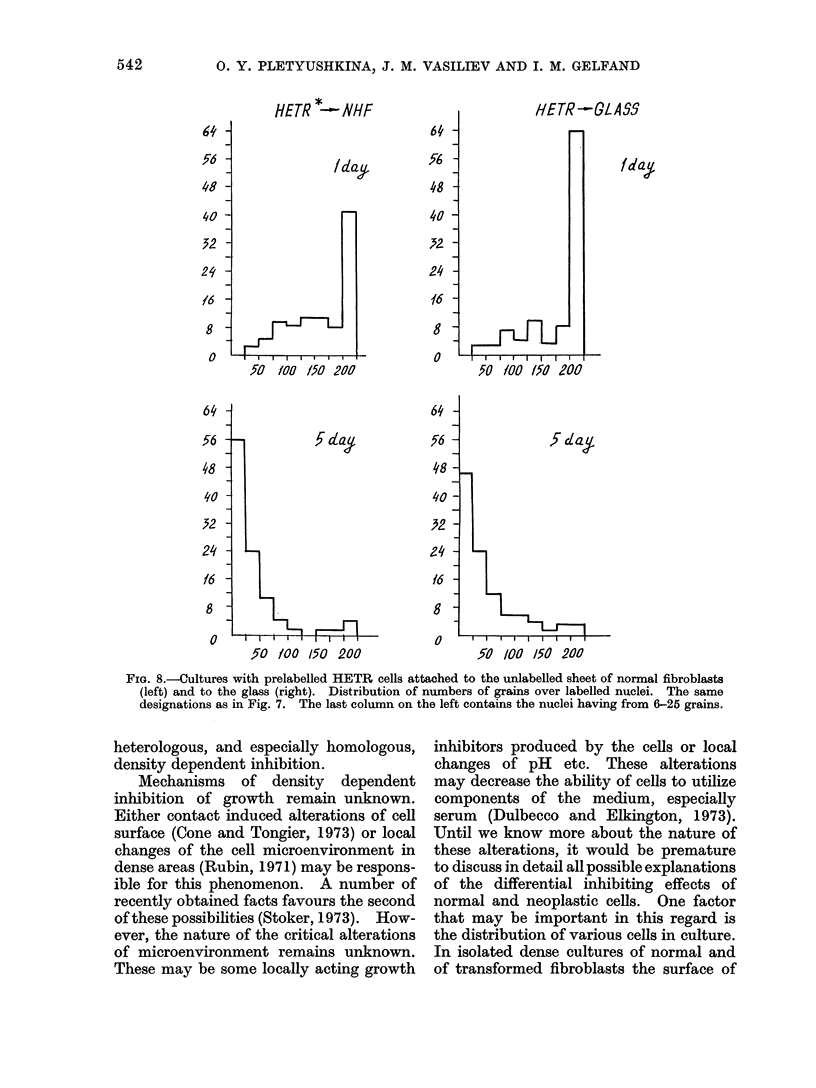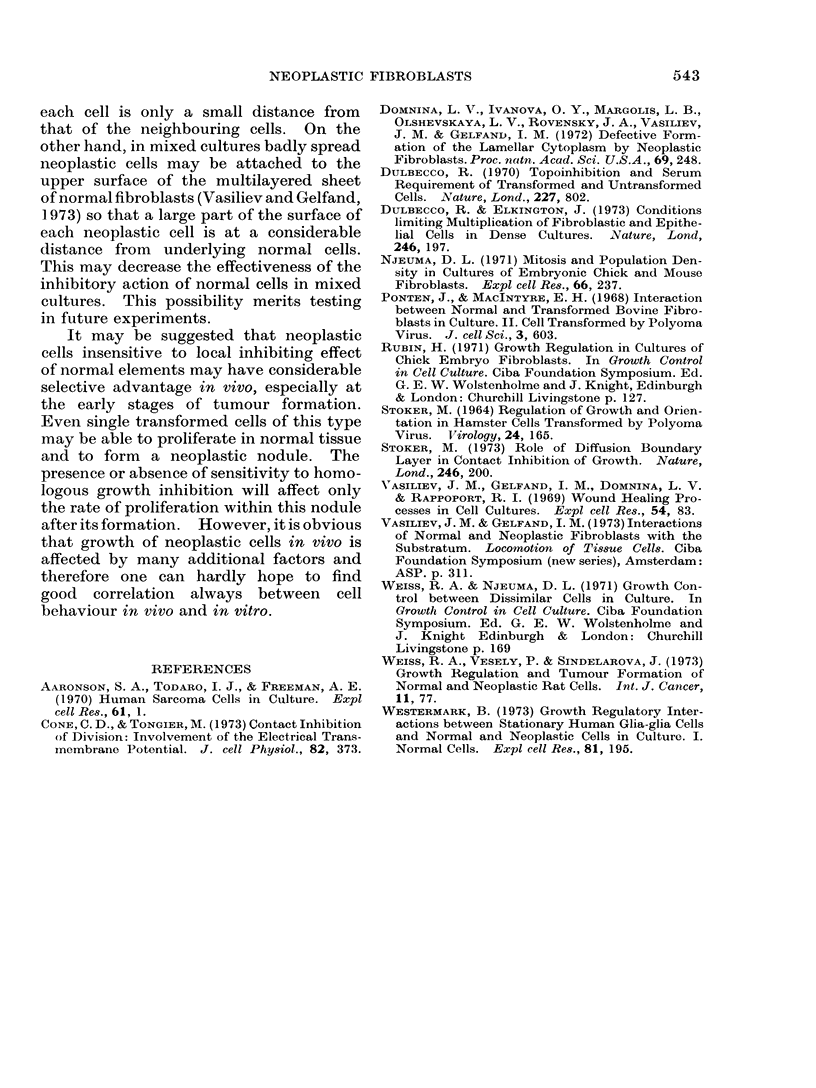# Neoplastic fibroblasts sensitive to the growth inhibition by homologous cells but insensitive to inhibition by parent normal cells.

**DOI:** 10.1038/bjc.1975.94

**Published:** 1975-05

**Authors:** O. Y. Pletyushkina, J. M. Vasiliev, I. M. Gelfand

## Abstract

3H-thymidine labelling and autoradiography were used to compare density dependent inhibition of growth in the cultures of two transformed lines of hamster fibroblasts and in primary cultures of their parent normal cells. Similar manifestations of density dependent inhibition were found in the isolated cultures of normal and neoplastic cells: at saturation densities these cultures had low labelling indices; these indices considerably increased when the cells migrated into the wound from the dense sheet, prelabelled cells seeded on the dense sheets of unlabelled homologous cells did not proliferate. However, proliferation of neoplastic cells was not inhibited when they were seeded on the dense sheet of normal fibroblasts. Thus, neoplastic hamster fibroblasts of both lines retained sensitivity to the inhibiting effect of homologous neoplastic cells but completely lost sensitivity to the inhibiting effect of normal fibroblasts. The possible significance of this selective loss of the sensitivity to normal cells is discussed briefly.


*Br. J. Cancer* (1975) **31**, 535

### NEOPLASTIC FIBROBLASTS SENSITIVE TO THE GROWTH INHIBITION BY HOMOLOGOUS CELLS BUT INSENSITIVE TO INHIBITION BY PARENT NORMAL CELLS

O. Y. PLETYUSHKINA, J. M. VASILIEV AND I. M. GELFAND

*From the Laboratory of Mathematical Biology of the Moscow State University and Institute of Experimental and Clinical Oncology of the Academy of Medical Sciences of USSR, Moscow, USSR*

Received 14 January 1975. Accepted 20 January 1975

**Summary—$^3$H-thymidine labelling and autoradiography were used to compare density dependent inhibition of growth in the cultures of two transformed lines of hamster fibroblasts and in primary cultures of their parent normal cells. Similar manifestations of density dependent inhibition were found in the isolated cultures of normal and neoplastic cells: at saturation densities these cultures had low labelling indices; these indices considerably increased when the cells migrated into the wound from the dense sheet, prelabelled cells seeded on the dense sheets of unlabelled homologous cells did not proliferate. However, proliferation of neoplastic cells was not inhibited when they were seeded on the dense sheet of normal fibroblasts. Thus, neoplastic hamster fibroblasts of both lines retained sensitivity to the inhibiting effect of homologous neoplastic cells but completely lost sensitivity to the inhibiting effect of normal fibroblasts. The possible significance of this selective loss of the sensitivity to normal cells is discussed briefly.**

INSENSITIVITY to the local inhibiting effect of homologous cells (loss of the homologous density dependent inhibition of growth) is usually regarded as one of the characteristic traits of transformed fibroblasts. At the same time, certain lines of transformed cells retain sensitivity to heterologous inhibition: their growth is inhibited by normal fibroblasts (Stoker, 1964; Ponten and MacIntyre, 1968; Weiss and Njeuma, 1971). Other transformed lines are insensitive to heterologous growth inhibition (Aaronson, Todaro and Freeman 1970; Weiss, Vesely and Sindelarova, 1973). Experiments described in this paper show that loss of homologous inhibition is not a prerequisite for the loss of heterologous one: transformed cells may completely lose sensitivity to the heterologous growth inhibition by normal cells while retaining sensitivity to the inhibition by homologous cells.

### MATERIALS AND METHODS

The cells of three types were used: (1) Normal embryo fibroblasts of golden hamster (NHF); these were first and second passages of the cells obtained by trypsinization of hamster embryos; (2) HETR line; these were spontaneously transformed hamster fibroblasts; (3) HEK-40 line; established from a hamster fibroblast culture treated with green monkey cytomegalovirus. These lines were established in the laboratory of immunology of the Institute of Experimental and Clinical Oncology and obtained from this laboratory by courtesy of Dr G. I. Deichman. At the time of the experiments both lines had been in culture for about 3 years. Morphologically the sparse cultures of HEK-40 and HETR lines consisted of polygonal and fusiform cells badly spread on the substrate; dense cultures consisted of multilayered sheets of badly orientated cells. The cells of these lines gave positive agglutination tests with Concanavalin A.

HETR cells were less oncogenic than HEK-40. Subcutaneous implantation of $10^6$ HETR cells to weanling hamsters produced palpable tumours in about 50% of animals 2 months after injection. The same number of HEK-40 cells produced tumours in 100% animals after 10 days.

The cells were grown in small (10 ml)

glass flasks, each flask containing one 20 $\times$ 10 $mm^2$ glass coverslip; $2 \times 10^5$ cells suspended in 2 ml of the medium were seeded in each flask. Two types of culture media were used. Medium I consisted of 45% of medium 199, 45% hydrolysate of lactalbumin and 10% of bovine serum. Medium II contained 45% of Eagle's medium in place of medium 199; and the other two components were the same. The medium was changed every 48 h. The wounds in cultures were made as described earlier (Vasiliev *et al.*, 1969).

Tritium-labelled thymidine ($^3$H-TdR, specific activity 1·3 Ci/mol; Sojuzizotop, USSR) was used in autoradiographic experiments. The cultures were fixed in a mixture of ethyl alcohol and acetic acid, then covered with fluid autoradiographic emulsion (VNIKFI USSR), exposed for 1–4 weeks, developed and stained with Mayer's haematoxylin. Two or more cultures were fixed in each group of one experiment. Each experiment was repeated two or more times.

The special control experiments were made in order to check whether autoradiography reveals all labelled cells even in dense multilayered cultures. $^3$H-TdR (0·1 $\mu$Ci/ml) was added to the fresh medium of HEK-40 and HETR cultures 4 days after seeding. The medium was changed 48 h later and $^3$H-TdR was added again. Cultures were fixed after 8 days of growth. Autoradiography revealed label in 96–99% of the nuclei of these cultures. Thus, there was no significant screening of labelled nuclei in the lower layers of cultures by the other cells located nearer to the emulsion.

Two main types of autoradiographic experiments were performed: experiments with pulse labelling and those with prelabelled cells seeded on the unlabelled sheets. In the experiments with pulse labelling $^3$H-TdR (0·5 $\mu$Ci/ml) was added to the medium 30 min before fixation of cultures. Labelling index (LI,% of labelled interphase nuclei) was determined by counting 1500–2000 cells in each culture.

To determine the dependence of LI on the local density of cell population the numbers of labelled and unlabelled nuclei were counted separately in each of the 400 randomly chosen fields of view of one culture. The area of one field was 0·007 $mm^2$. Labelling indices in all fields having similar cell densities were then calculated (see Fig. 2).

To assess the growth of prelabelled cells on the established sheet of unlabelled cells, we used the method of Stoker (1964) with the following modification. The cells were labelled by growing for 4 days in the medium containing $^3$H-TdR(0·1 $\mu$Ci/ml) and then suspended. Six-day old cultures were used as unlabelled cell sheets. At the beginning of the experiment one half of each sheet was removed using a razor blade. The medium was then changed; a suspension of prelabelled cells ($5 \times 10^4$ cells/flask) was added to the fresh medium. To prevent reutilization of label, unlabelled thymidine (2·5 $\mu$g/ml) was added to this medium and to those used for the following medium changes. The cultures were fixed 1, 3 or 5 days after the addition of prelabelled cells and autoradiographic preparations were made. The number of labelled cells per unit area of the sheet and of the glass surface was counted in these preparations. A total of 100 fields of view were counted in each part of the culture; the area of one field was 0·01 $mm^2$. The counted areas on the sheet and on the glass were chosen randomly at distances not less than 2 mm from the wounded edge of the sheet. All nuclei that had 6 or more silver grains over them were counted as labelled; background labelling was less than 1 grain over each unlabelled nucleus. At Day I after seeding 95–99% of cells attached to the glass surfaces were labelled; at Day 5 87–95% cells on the glass still remained labelled. Thus, dilution of label in the course of cell multiplication was not sufficient to decrease significantly the proportion of the prelabelled cells that could be revealed autoradiographically. To assess the dilution of label caused by cell division the numbers of silver grains over 50–100 labelled nuclei were counted in several experiments and histograms of the distribution of these nuclei were made.

### RESULTS

### *Experiments with isolated cultures of normal and transformed fibroblasts*

The cultures of all 3 cell types grew to certain saturation densities (Table, Fig. 1). In medium I the saturation density of HEK-40 cells was twice as high as that of NHF; HETR had intermediate values of saturation densities. In medium II NHF grew to higher saturation densities than in medium I. In contrast, satur-

TABLE.—*Saturation Densities of Cell Populations in the Cultures of Normal and Transformed Hamster Fibroblasts*

| Culture | Saturation density × $10^{-5}$ (cell/cm$^2$) Medium I | Medium II |
|---|---|---|
| Embryo hamster fibroblasts | 5·0–5·5 | 6·0–8·0 |
| HETR line | 6·5–7·0 | 7·0–7·5 |
| HEK-40 line | 8·0–11·0 | 8·0–11·0 |

FIG. 1.—Proliferation of isolated cultures of normal hamster embryo fibroblasts (NHF) and of transformed fibroblasts (lines HETR and HEK-40) in medium I. Solid lines show mean labelling indices in pulse labelled cultures (left ordinates). Interrupted lines show mean population densities ($\times 10^{-5}$ cells per cm$^2$ of the substrate) in the same cultures (right ordinates). Abscissa is time of cultivation (days after seeding). Medium changes are shown by arrows. Results of one experiment are shown for each cell type.

ation densities of transformed HEK-40 cells were similar in media I and II. HETR cells were similar in media I and II. HETR had slightly higher saturation density in medium II. As a result, differences between saturation densities of normal and transformed cells were much smaller in medium II than in medium I.

Manifestations of the density-dependent inhibition of growth were seen in the cultures of all 3 cell types grown in both variants of media: (a) LI determined in cultures pulse labelled at 48 h after each medium change gradually decreased as time of growth and mean population density increased (Fig. 1); (b) in individual dense cultures LI were measured in areas with various cell densities (see Methods). In cultures of each of the 3 cell types LI decreased as local cell density increased (Fig. 2). For areas with similar densities cultures of NHF grown in medium I had the lowest LI and cultures of HEK-40 the highest one. These differences between cultures of various cells were much less pronounced in medium II; (c) migration into the wound from dense cell sheet considerably increased LI (Fig. 3). Migration from sparse 2-day old cultures did not affect LI; (d) prelabelled cells seeded on the dense sheet of homologous unlabelled cells did not multiply on this sheet: the density of labelled cells on the sheet did not increase between 1 and 5 days after seeding (Fig. 4, 5, 6). In contrast, the density of labelled cells attached to the glass in the same cultures increased considerably. Thus, cell divisions took place on the glass but not on the sheet. This conclusion was confirmed by the counts of the numbers of grains over labelled nuclei (Fig. 7). On the glass modal number of grains at Day 5 decreased considerably compared with Day 1, indicating that several divisions of most labelled cells took place. No dilution of label was seen on the sheet.

*Experiments with mixed cultures*

In these experiments labelled neoplastic cells were seeded on the normal

FIG. 2.—Labelling indices (ordinates) in areas with different local cell population densities (abscissa is number of cells per field of the microscope) of dense isolated cultures of various cell types. Each line was obtained by counts made in one culture; see text for details. Cultures were grown in medium I and fixed after 6 days of growth, 48 h after the last medium change. The mean LI in these cultures were: 9·6 (NHF); 3·4 (HETR); 7·4 (HEK-40). Vertical bars are standard errors.

FIG. 3.—Mean labelling indices (ordinates) in wounded cultures of NHF and of HEK-40. The cultures were grown in medium I. They were pulse labelled and fixed 24 h after wounding; the last medium change was made 24 h before wounding. Abscissas are days of growth at the moment of fixation. Solid lines show mean LI in the wound, interrupted lines, LI in the dense cell sheet.

FIG. 4.—Proliferation of prelabelled normal hamster fibroblasts on the unlabelled sheet of homologous cells and on the glass in the same cultures. Ordinate is the mean number of labelled cells per 1 mm² of the sheet (interrupted line) and of the glass (solid line). Abscissa is time after seeding of labelled cells (days). Cultures were grown in medium II. The unlabelled culture was 6 days old at the moment of seeding of labelled cells.

cell sheet; 24 h after seeding, the densities of labelled cells on the sheet and on the glass were similar (Fig. 5, 6). In the following days the density of labelled cells on the sheet increased at the same, or almost the same, rate as that on the glass. In a number of experiments the same suspension of labelled transformed fibroblasts was seeded on the sheets of homologous transformed cells and on the sheets of normal cells. Medium II was used in these experiments so that the sheets of both types had similar population densities. In all experiments densities of labelled transformed cells increased between 1 and 5 days on the glass and on the normal sheet but not on the homologous sheets (Fig. 5, 6). Dilution of label between 1 and 5 days was similar on the glass and on the normal cell sheet (Fig. 8). Thus, division of labelled transformed cells was not inhibited on the sheet of normal cells. When prelabelled normal cells were seeded in the cultures containing dense sheets of unlabelled transformed fibroblasts, cell attachment and multiplication were poor both on the glass and on the sheets in these cultures. Therefore, the effect of the sheet of transformed cells on the proliferation of normal fibroblasts could not be adequately assessed.

### DISCUSSION

Fibroblasts of HETR and HEK-40 lines have characteristic properties of transformed cells, including altered morphology and oncogenicity. Experiments made by one of us (O.P.) indicate that wound serum requirement measured according to Dulbecco (1970) is much lower for HEK-40 and HETR than for NHF. Saturation density and oncogenicity of HEK-40 are somewhat higher than that of HETR. In certain conditions of culture (medium I) saturation densities of transformed cells are higher than those of normal fibroblasts. However, in other media these differences were diminished. In both media when the cultures of transformed cells were near their saturation densities all the manifestations of density dependent inhibition of growth could be seen; these manifestations were similar to those seen in dense normal cultures. In particular, it is important to stress that growth inhibition in dense culture of normal and transformed cells is dependent on local factors. This inhibition is observed only in those areas of cultures where local density of cell population is high; inhibition is absent in other areas of the same cultures where cell population is sparse. This is clearly shown by the results of the experiments with wounded cultures. The same conclusion is confirmed by the comparison of the proliferation of prelabelled cells on

FIG. 5.—Proliferation of prelabelled HETR cells in the unlabelled cultures of homologous cells (left, HETR* - - - HETR) and of normal fibroblasts (right, HETR* - - - NHF). One suspension of prelabelled cells was used for seeding in the unlabelled cultures of both types. All designations and conditions of the experiment are the same as in Fig. 4.

FIG. 6.—Proliferation of prelabelled HEK-40 cells in the unlabelled cultures of homologous cells (left, HEK-40* - - - HEK-40) and of normal fibroblasts (right, HEK-40* - - - NHF). All designations and conditions of the experiment are the same as in Fig. 4 and 5.

the homologous cell sheet and on the glass in the same culture.

Although the transformed HETR and HEK-40 fibroblasts were sensitive to local inhibiting effect of homologous cells, they were not inhibited by the parent normal cells. This different sensitivity was observed in the experiments with transformed fibroblasts seeded on normal and neoplastic cell sheets having similar population density, that is, it was dependent on the nature of interacting cells. In future it would be worthwhile to compare mutual effects of various neoplastic cells in mixed cultures.

These data show that in the course of

Fig. 7.—Cultures with prelabelled HETR cells attached to the unlabelled homologous cell sheet (left) and to the glass (right). Histograms of the distribution of numbers of grains over labelled nuclei at 1 day (above) and at 5 days (below) after seeding. Ordinates are % of nuclei with given numbers of grains. Abscissas are number of grains over one nucleus. Each column shows % nuclei having the number of grains ranging from that shown by the left abscissa of this column to that shown by the right one. The last column on the left contains the nuclei having from 6 to 20 grains. The last column on the right contains all the nuclei having more than 200 grains.

neoplastic transformation certain cell lines may lose sensitivity to the inhibiting effect of parent normal cells but not to homologous neoplastic cells. Similar data were obtained for transformed mouse fibroblasts of the L line (Domnina *et al.*, 1972). In the experiments of Weiss *et al.* (1973), one of the established rat cell lines (LW13) retained a moderate degree of homologous growth inhibition but was not inhibited by normal fibroblasts.

It should be added that other variants of differential sensitivity of cells to the homologous and heterologous growth inhibition have been described in the literature: (1) Normal cells may be sensitive to the inhibiting effect of homologous normal cells but not to that of certain heterologous normal cells of other origin (Njeuma, 1971; Westermark, 1973); (2) certain neoplastic cells may retain sensitivity to the inhibiting effect of parent normal cells but lose sensitivity to homologous cells (Stoker, 1964; Ponten and Macintyre, 1968; Weiss and Njeuma, 1971). The properties of cells described by this second group of workers seem to be complementary to those of the cells reported in this paper: possibly, certain transformed lines lose sensitivity only to homologous cells but not to normal ones, while others, alternatively, become insensitive only to normal cells. However, it would be premature to make any generalizations as somewhat different criteria were used in various papers to judge

FIG. 8.—Cultures with prelabelled HETR cells attached to the unlabelled sheet of normal fibroblasts (left) and to the glass (right). Distribution of numbers of grains over labelled nuclei. The same designations as in Fig. 7. The last column on the left contains the nuclei having from 6–25 grains.

heterologous, and especially homologous, density dependent inhibition.

Mechanisms of density dependent inhibition of growth remain unknown. Either contact induced alterations of cell surface (Cone and Tongier, 1973) or local changes of the cell microenvironment in dense areas (Rubin, 1971) may be responsible for this phenomenon. A number of recently obtained facts favours the second of these possibilities (Stoker, 1973). However, the nature of the critical alterations of microenvironment remains unknown. These may be some locally acting growth inhibitors produced by the cells or local changes of pH etc. These alterations may decrease the ability of cells to utilize components of the medium, especially serum (Dulbecco and Elkington, 1973). Until we know more about the nature of these alterations, it would be premature to discuss in detail all possible explanations of the differential inhibiting effects of normal and neoplastic cells. One factor that may be important in this regard is the distribution of various cells in culture. In isolated dense cultures of normal and of transformed fibroblasts the surface of each cell is only a small distance from that of the neighbouring cells. On the other hand, in mixed cultures badly spread neoplastic cells may be attached to the upper surface of the multilayered sheet of normal fibroblasts (Vasiliev and Gelfand, 1973) so that a large part of the surface of each neoplastic cell is at a considerable distance from underlying normal cells. This may decrease the effectiveness of the inhibitory action of normal cells in mixed cultures. This possibility merits testing in future experiments.

It may be suggested that neoplastic cells insensitive to local inhibiting effect of normal elements may have considerable selective advantage *in vivo*, especially at the early stages of tumour formation. Even single transformed cells of this type may be able to proliferate in normal tissue and to form a neoplastic nodule. The presence or absence of sensitivity to homologous growth inhibition will affect only the rate of proliferation within this nodule after its formation. However, it is obvious that growth of neoplastic cells *in vivo* is affected by many additional factors and therefore one can hardly hope to find good correlation always between cell behaviour *in vivo* and *in vitro*.

### REFERENCES

AARONSON, S. A., TODARO, I. J., & FREEMAN, A. E. (1970) Human Sarcoma Cells in Culture. *Expl cell Res.*, **61**, 1.

CONE, C. D., & TONGIER, M. (1973) Contact Inhibition of Division: Involvement of the Electrical Transmembrane Potential. *J. cell Physiol.*, **82**, 373.

DOMNINA, L. V., IVANOVA, O. Y., MARGOLIS, L. B., OLSHEVSKAYA, L. V., ROVENSKY, J. A., VASILIEV, J. M. & GELFAND, I. M. (1972) Defective Formation of the Lamellar Cytoplasm by Neoplastic Fibroblasts. *Proc. natn. Acad. Sci. U.S.A.*, **69**, 248.

DULBECCO, R. (1970) Topoinhibition and Serum Requirement of Transformed and Untransformed Cells. *Nature, Lond.*, **227**, 802.

DULBECCO, R. & ELKINGTON, J. (1973) Conditions limiting Multiplication of Fibroblastic and Epithelial Cells in Dense Cultures. *Nature, Lond*, **246**, 197.

NJEUMA, D. L. (1971) Mitosis and Population Density in Cultures of Embryonic Chick and Mouse Fibroblasts. *Expl cell Res.*, **66**, 237.

PONTEN, J., & MACINTYRE, E. H. (1968) Interaction between Normal and Transformed Bovine Fibroblasts in Culture. II. Cell Transformed by Polyoma Virus. *J. cell Sci.*, **3**, 603.

RUBIN, H. (1971) Growth Regulation in Cultures of Chick Embryo Fibroblasts. In *Growth Control in Cell Culture*. Ciba Foundation Symposium. Ed. G. E. W. Wolstenholme and J. Knight, Edinburgh & London: Churchill Livingstone p. 127.

STOKER, M. (1964) Regulation of Growth and Orientation in Hamster Cells Transformed by Polyoma Virus. *Virology*, **24**, 165.

STOKER, M. (1973) Role of Diffusion Boundary Layer in Contact Inhibition of Growth. *Nature, Lond.*, **246**, 200.

VASILIEV, J. M., GELFAND, I. M., DOMNINA, L. V. & RAPPOPORT, R. I. (1969) Wound Healing Processes in Cell Cultures. *Expl cell Res.*, **54**, 83.

VASILIEV, J. M. & GELFAND, I. M. (1973) Interactions of Normal and Neoplastic Fibroblasts with the Substratum. *Locomotion of Tissue Cells*. Ciba Foundation Symposium (new series), Amsterdam: ASP. p. 311.

WEISS, R. A. & NJEUMA, D. L. (1971) Growth Control between Dissimilar Cells in Culture. In *Growth Control in Cell Culture*. Ciba Foundation Symposium. Ed. G. E. W. Wolstenholme and J. Knight Edinburgh & London: Churchill Livingstone p. 169

WEISS, R. A., VESELY, P. & SINDELAROVA, J. (1973) Growth Regulation and Tumour Formation of Normal and Neoplastic Rat Cells. *Int. J. Cancer*, **11**, 77.

WESTERMARK, B. (1973) Growth Regulatory Interactions between Stationary Human Glia-glia Cells and Normal and Neoplastic Cells in Culture. I. Normal Cells. *Expl cell Res.*, **81**, 195.